# The prevalence of phantom limb pain and associated risk factors in people with amputations: a systematic review protocol

**DOI:** 10.1186/s13643-018-0938-8

**Published:** 2019-01-10

**Authors:** Katleho Limakatso, Gillian J. Bedwell, Victoria J. Madden, Romy Parker

**Affiliations:** 1 Department of Anaesthesia and Perioperative Medicine, University of Cape Town, Cape Town, South Africa; 2 Department of Health and Rehabilitation Sciences, Division of Physiotherapy, University of Cape Town, Cape Town, South Africa; 3 Department of Psychiatry and Mental Heath, University of Cape Town, Cape Town, South Africa

**Keywords:** Prevalence, Risk factors, Phantom limb pain, Amputations

## Abstract

**Background:**

The prevalence of phantom limb pain (PLP) in people with amputations is unclear because of the conflicting reports across the literature. It is proposed that the conflicting reports on the prevalence of PLP are a consequence of variations in the time period during which the studies were undertaken, countries in which the studies were conducted and recruitment processes implemented during collection of epidemiological data. In consideration of these factors, we aim to gather and critically appraise relevant literature to determine the prevalence estimate of and risk factors for PLP in people with amputations.

**Methods:**

We will use a customised search strategy containing relevant words and terms to search the following databases: MEDLINE/PubMed (via EBSCOhost), PsycINFO (via EBSCOhost), PsycArticles, Cumulative Index to Nursing and Allied Health Literature (CINAHL) (via EBSCOhost), Africa-Wide Information (via EBSCOhost), Health Source: Nursing/Academic Edition (via EBSCOhost) SCOPUS, Web of Science and Academic Search Premier (via EBSCOhost). The risk of bias assessment will be conducted using a risk of bias assessment tool for prevalence studies, and data will be extracted using a piloted customised data extraction sheet. Data extracted from individual studies will be entered into Review Manager 5 and assessed for clinical and statistical heterogeneity. Studies will be pooled for meta-analysis using the random-effects model to determine a summary estimate of the prevalence of PLP across included studies. A statistically significant level will be set at *p* < 0.05.

**Discussion:**

As far as we know, a systematic review and meta-analysis on the prevalence of, and risk factors for PLP in people with amputations has not been conducted. Given the varying reports in the literature, it is necessary to determine an estimate of the prevalence of PLP to generate an informed conclusion on this subject. The results of this review will be published in an internationally accredited journal and used to inform researchers, clinicians, policy-makers and the public about the burden of, and risk factors for PLP. This will be done with a further aim to improve the quality of pain management in society.

**Systematic review registration:**

PROSPERO CRD42018094821

**Electronic supplementary material:**

The online version of this article (10.1186/s13643-018-0938-8) contains supplementary material, which is available to authorized users.

## Background

Phantom limb pain (PLP) is defined as pain felt in the missing portion of the amputated limb following amputation [1]. Phantom limb pain commonly occurs in people with limb amputations due to trauma or surgery. However, some cases of PLP have been reported in congenital amputees [2, 3]. It has been proposed that risk factors such as persisting pre-operative pain, stump pain and time period since amputation contribute to the onset of PLP [4]. Phantom limb pain remains a serious public health problem because it is common and often undertreated [5]. As a result, persisting PLP may contribute further to depression and problems with prosthesis use, sleep and participation in activities of daily function [1, 6, 7].

The prevalence of PLP among people with amputation is unclear, perhaps because of conflicting reports across the literature. While some studies report a high prevalence of 85% [8, 9], one study has reported a markedly lower prevalence of 33% [10]. The conflicting reports in the literature regarding the prevalence of PLP is perplexing. It has been proposed that such discrepancy is likely caused by a variation in the time period during which the studies were undertaken, the countries in which the studies were conducted and the recruitment processes implemented during collection of epidemiological data [11].

Early studies, conducted during a period when PLP was commonly characterised as a psychiatric disorder, reported low prevalence rates [12]. Perhaps low rates could be accounted for by the patients’ fear of reporting their pain to avoid the stigma associated to PLP. Low prevalence rates are also recorded in studies conducted in developing countries where the stigma associated with chronic pain conditions is still common [13]. Finally, many prevalence studies of PLP were conducted in clinical settings using patients continuing with medical care, introducing a recruitment bias [10, 14–17]. Thus, patients without access to clinical care may be unaccounted for in these statistics.

Prevalence studies are key to informing researchers, clinicians, policy-makers and the public about the burden of disease in society [18]. However, a wide variation in the reported prevalence of PLP does not provide a definitive prevalence estimate, and therefore hinders the development or implementation of effective interventions for preventing or treating PLP. Further, up to date, there is no systematic review that has synthesised data on the risk factors for PLP. To address this gap in the literature, a systematic review and meta-analysis will be conducted by gathering and critically appraising relevant literature regarding the prevalence of, and risk factors for PLP in people with amputations. The results of this review will enable us to generate an evidence-informed conclusion on the prevalence estimate of PLP, as well as associated risk factors in people with amputations. Further, they will be used to motivate for the development and implementation of pragmatic interventions that may prevent or reduce PLP in people with amputation. The current evidence suggests that rehabilitation approaches rather than pharmacological approaches are most effective for treating PLP [19]. Therefore, the results of this review may also highlight the need for enough access not only to medication but also to physiotherapists and occupational therapists for the treatment of PLP.

## Objective

The purpose of this review will be to determine the prevalence estimate of and risk factors for PLP in people with amputations. In addition, this review will determine if there is an association between the prevalence of PLP and the development status (developed vs developing) of countries in which the studies were conducted.

## Methods

This protocol was developed in accordance with the Preferred Reporting Items of Systematic Reviews and Meta-Analysis Protocol (PRISMA-P) guidelines [20], and has been registered on PROSPERO—an international prospective register of systematic reviews with health-related outcome [21]. The PRISMA-P guidelines fulfilled by this protocol are presented in Additional file 1.

### Criteria for selecting studies for this review

#### Inclusion criteria

- Study design and participants: published and unpublished prevalence case-control, cross-sectional and cohort studies on PLP in surgical, traumatic and congenital amputees aged ≥ 18 years.
- Outcome: prevalence of PLP and/or risk factors for PLP.
- Study setting: clinical and community-based studies conducted worldwide.
- Language of publication: studies published in the English language.
- Years: 1980–2018

#### Exclusion criteria

- Intervention (only) studies.

### Search strategy for identification of studies

#### Electronic searches

One investigator (KL) and a senior medical librarian will use a customised search strategy (Appendix) containing appropriate words and terms to search the following databases: MEDLINE/PubMed (via EBSCOhost), PsycINFO (via EBSCOhost), PsycArticles, Cumulative Index to Nursing and Allied Health Literature (CINAHL) (via EBSCOhost), Africa-Wide Information (via EBSCOhost), Health Source: Nursing/Academic Edition (via EBSCOhost) SCOPUS, Web of Science and Academic Search Premier (via EBSCOhost). Studies identified from this electronic search will be saved on EndNote X8 programme, which will also be used to remove duplicates [22].

#### Search of other sources

We will search the reference list of all eligible studies to identify additional studies with the potential for inclusion in this review. To identify grey literature, we will search OpenGrey www.opengrey.eu, and contact experts on ResearchGate www.researchgate.net to seek unpublished, and ongoing studies that may be eligible for inclusion.

### Data collection and analysis

#### Study screening

Following the removal of duplicates, retained studies will be transferred to the Covidence systematic review software available at www.covidence.org. This software will be used as an online collaboration platform for reviewers during the entire screening process. Two reviewers (KL and GJB) will independently screen study titles and abstracts for eligibility. Two reviewers (KL and GJB) will independently assess full-text articles retained from the initial screening for eligibility using the inclusion and exclusion criteria. The entire review process will be illustrated using a PRISMA flowchart detailing included studies and excluded studies, with reasons for their exclusion.

#### Data extraction and management

Two reviewers (KL and GJB) will independently extract data using a piloted customised data extraction sheet. The following data will be extracted: authors, year of publication, study setting, country of study, sample size, participants’ age and gender, site of amputation, PLP prevalence and risk factors, and author conflict of interest statement. Completed data extraction forms will be stored on a password-protected online storage platform which will be accessible only to the reviewers. Any disagreements between reviewers will be resolved by discussion. A third reviewer (RP) will be consulted if a consensus cannot be reached.

#### Risk of bias assessment

Two reviewers (KL and GJB) will independently conduct a risk of bias assessment using a risk of bias assessment tool for prevalence studies developed by Hoy et al. [23]. The results of this assessment will be classified as either low, moderate or high risk. Any disagreements between reviewers will be resolved by discussion. A third reviewer (VJM) will be consulted if a consensus cannot be reached.

#### Data analysis

Data extracted from individual studies will be entered into Review Manager 5 [24] for analysis. Clinical heterogeneity will be determined based on similarities or differences in participant and outcome characteristics, recruitment procedures and study setting [25]. Statistical heterogeneity will be assessed using the *I*^2^ statistic, and the results will be presented as low (< 25%), moderate (25–50%) and high (> 50%) [26]. Subject to consideration of heterogeneity and risk of bias, studies will be pooled for meta-analysis using the random-effects model to determine a summary estimate of PLP prevalence across included studies. A statistically significant level will be set at *p* < 0.05. A narrative data analysis will be conducted where there is insufficient data to conduct a meta-analysis. A funnel plot will be generated to assess for possible publication bias [27]. In addition, the Egger’s regression test will be used to assess for the asymmetry of the funnel plot. A significant result (*p* < 0.05) will indicate a possible publication bias [27]. Risk factors for PLP will be identified from included studies and synthesised descriptively. Cohen’s Kappa will be used to determine inter-rater agreement during screening, data extraction and risk of bias assessment as either minimal (0–0.39), weak (0.40–0.59), substantial (0.60–0.79) or strong (0.80–0.90) [28].

#### Subgroup analysis

A subgroup analysis on the prevalence of PLP will be conducted based on the development status of the countries in which the studies were conducted. Each country will be allocated to either group (developing vs developed) using the World Economic Situation and Prospects (WESP) classification system [29]. Vascular complications are the common cause of amputations in developed countries and traumatic accidents are the common cause of amputations in developing countries [30–32]. Regardless of the differences in the reasons for amputations, recent evidence shows that there is a similar number of limb amputations conducted in developing and developed countries [33, 34]. However, it is unclear whether there is a difference in the reported prevalence of PLP between developing and developed countries. The purpose of this subgroup analysis is to determine if this is the case.

### Grading the certainty of evidence

The Grading of Recommendations Assessment, Development, and Evaluation (GRADE) methodology will be used to determine the certainty of evidence regarding the prevalence of PLP. The quality of evidence will be graded as high if further research is unlikely to change the effect estimates, moderate if further research is likely to have a considerable impact on the effect estimates and low if further research is likely to be capable of changing the effect estimates [35].

### Dealing with missing data

We will contact the authors of included studies to request missing data as necessary. If additional data cannot be obtained, each study with incomplete data will not be analysed.

## Discussion

As far as we know, a systematic review and meta-analysis on the prevalence of and risk factors for PLP in people with amputations has not been conducted. Given the conflicting reports in the literature concerning the prevalence of PLP, it is necessary to determine the estimate of the prevalence of PLP to generate an informed conclusion on this subject. The results of this review will be published in a peer-reviewed journal and used to inform researchers, clinicians, policy-makers and the public about the burden of PLP in society. Further, they will be used to motivate for the development and implementation of pragmatic interventions that could prevent or reduce PLP in people with amputation. By clarifying risk factors for PLP, this study will provide empirical evidence that may enable clinicians to identify priorities for diagnosing, treating and preventing PLP.

### Additional file

Additional file 1: PRISMA-P Checklist. (DOCX 30 kb)

## Appendix

### Search strategy [PubMed]

1. Amputation [MeSH] OR Amputation, Traumatic [MeSH] OR Amputation Stumps [MeSH] OR Amputee OR amputees OR amputation OR limb deficiency OR limb loss.
2. Phantom Limb [MeSH] OR Phantom limb OR phantom pain OR phantom sensations OR phantom sensation OR residual limb pain
3. Epidemiology [MeSH] OR Epidemiology [Subheading] OR Prevalence [MeSH] OR Risk Factors [MeSH]
4. associated OR association OR burden OR case-control OR cohort OR correlation OR correlates OR cross-sectional OR determinant OR epidemiology OR epidemiological OR epidemiologic OR frequency OR incidence OR interview OR likelihood ratio OR observational OR occur OR occurrence OR odds ratios OR predict OR predictor OR prediction OR present OR presentation OR prevalence OR prevalent OR probability OR prognosis OR prognostic OR proportion OR prospective OR questionnaire OR questionnaires OR rate OR retrospective OR risk OR risks OR self-report OR statistic OR surveillance OR survey OR surveys
5. 1 AND 2 AND 3 AND 4

## Abbreviations

CINAHL: Cumulative Index to Nursing and Allied Health Literature
GRADE: Grading of Recommendations Assessment, Development, and Evaluation
PLP: Phantom limb pain
PRISMA-P: Preferred Reporting Items of Systematic Reviews and Meta-Analysis Protocol

## References

[1] Ehde DM, Czerniecki JM, Smith DG, Campbell KM, Edwards WT, Jensen MP (2000). Chronic phantom sensations, phantom pain, residual limb pain, and other regional pain after lower limb amputation. Arch Phys Med Rehabil.

[2] Melzack R, Israel R, Lacroix R, Schultz G (1997). Phantom limbs in people with congenital limb deficiency or amputation in early childhood. Brain J Neurol.

[3] Saadah E, Melzack R (1994). Phantom limb experiences in congenital limb-deficient adults. Cortex.

[4] Dijkstra PU, Geertzen JH, Stewart R, van der Schans CP (2002). Phantom pain and risk factors: a multivariate analysis. J Pain Symptom Manage.

[5] Alviar M, Hale T, Dungca M (2011). Pharmacologic interventions for treating phantom limb pain. Cochrane Database Syst Rev.

[6] Padovani MT, Martins MRI, Venâncio A, Forni JEN (2015). Anxiety, depression and quality of life in individuals with phantom limb pain. Acta Ortop Bras.

[7] Morgan SJ, Friedly JL, Amtmann D, Salem R, Hafner BJ (2017). Cross-sectional assessment of factors related to pain intensity and pain interference in lower limb prosthesis users. Arch Phys Med Rehabil.

[8] Sherman RA, Sherman CJ (1983). Prevalence and characteristics of chronic phantom limb pain among American veterans: results of a trial survey. Am J Phys Med Rehab.

[9] Parkes CM (1973). Factors determining the persistence of phantom pain in the amputee. J Psychosom Res.

[10] Ahmed A, Bhatnagar S, Mishra S, Khurana D, Joshi S, Ahmad SM (2017). Prevalence of phantom limb pain, stump pain, and phantom limb sensation among the amputated cancer patients in India: a prospective, observational study. Indian J Palliat Care.

[11] Maimela E, Alberts M, Modjadji SE, Choma SS, Dikotope SA, Ntuli TS (2016). The prevalence and determinants of chronic non-communicable disease risk factors amongst adults in the Dikgale health demographic and surveillance system (HDSS) site, Limpopo Province of South Africa. PLoS One.

[12] Jensen TS, Krebs B, Nielsen J, Rasmussen P (1985). Immediate and long-term phantom limb pain in amputees: incidence, clinical characteristics and relationship to pre-amputation limb pain. Pain.

[13] Mishra S, Bhatnagar S, Gupta D, Diwedi A (2008). Incidence and management of phantom limb pain according to World Health Organization analgesic ladder in amputees of malignant origin. Am J Hos Palliat Med.

[14] Byrne KPA (2011). Survey of phantom limb pain, phantom sensation and stump pain in Cambodian and New Zealand amputees. Pain Med.

[15] Sherman RA, Sherman CJ, Gall NG (1980). A survey of current phantom limb pain treatment in the United States. Pain.

[16] Desmond DM, MacLachlan M (2010). Prevalence and characteristics of phantom limb pain and residual limb pain in the long term after upper limb amputation. Int J Rehabil Res.

[17] Ventham N, Heyburn P, Huston N (2010). 374 prevalence of phantom limb pain in diabetic and non-diabetic leg amputees: a cross-sectional observational survey. Eur J Pain Suppl.

[18] Moloi AH, Watkins D, Engel ME, Mall S, Zühlke L (2016). Epidemiology, health systems and stakeholders in rheumatic heart disease in Africa: a systematic review protocol. BMJ Open.

[19] Richardson C, Kulkarni J (2017). A review of the management of phantom limb pain: challenges and solutions. J Pain Res.

[20] Shamseer L, Moher D, Clarke M, Ghersi D, Liberati A, Petticrew M, Group, Prisma-P (2015). Preferred reporting items for systematic review and meta-analysis protocols (PRISMA-P) 2015: elaboration and explanation. BMJ.

[21] Sideri S, Papageorgiou SN, Eliades T. Registration in the international prospective register of systematic reviews (PROSPERO) of systematic review protocols was associated with increased review quality. J Clin Epidemiol. 2018;100:103–10. 10.1016/j.jclinepi.2018.01.003 29339215

[22] Rathvon D (2017). EndNote X8--citation manager--What's new?.

[23] Hoy D, Brooks P, Woolf A, Blyth F, March L, Bain C (2012). Assessing risk of bias in prevalence studies: modification of an existing tool and evidence of interrater agreement. J Clin Epidemiol.

[24] The Nordic Cochrane Centre. The Cochrane collaboration (2011). Review manager (RevMan). 5.1.

[25] Gagnier JJ, Moher D, Boon H, Beyene J, Bombardier C (2012). Investigating clinical heterogeneity in systematic reviews: a methodologic review of guidance in the literature. BMC Med Res Methodol.

[26] Higgins JP, Thompson SG, Deeks JJ, Altman DG (2003). Measuring inconsistency in meta-analyses. BMJ: Br Med J.

[27] Egger M, Smith GD, Schneider M, Minder C (1997). Bias in meta-analysis detected by a simple, graphical test. BMJ.

[28] Cohen J (1960). A coefficient of agreement for nominal scales. Educ Psychol Meas.

[29] Data sources, country classifications and aggregation methodology. (2014, January 25). Retrieved 4 Dec 2018, from http://www.un.org/en/development/desa/policy/wesp/wesp_current/2014wesp_country_classification.pdf.

[30] Rommers G, Vos L, Groothoff J, Schuiling C, Eisma W (1997). Epidemiology of lower limb amputees in the north of the Netherlands: aetiology, discharge destination and prosthetic use. Prosthetics Orthot Int.

[31] Unwin N (2000). Epidemiology of lower extremity amputation in centres in Europe, North America and East Asia. Br J Surg.

[32] Esquenazi A (2004). Amputation rehabilitation and prosthetic restoration. From surgery to community reintegration. Disabil Rehabil.

[33] Godlwana L, Nadasan T, Puckree T (2008). Global trends in incidence of lower limb amputation: a review of the literature. South Afr J Physiotherapy.

[34] Staats T (1996). The rehabilitation of the amputee in the developing world: a review of the literature. Prosthetics Orthot Int.

[35] Guyatt GH, Oxman AD, Vist GE, Kunz R, Falck-Ytter Y, Alonso-Coello P (2008). GRADE: an emerging consensus on rating quality of evidence and strength of recommendations. BMJ.

